# Perspectives and practices of healthcare providers and caregivers on healthcare-associated infections in the neonatal intensive care units of two hospitals in Ghana

**DOI:** 10.1093/heapol/czaa102

**Published:** 2020-11-09

**Authors:** Gifty Sunkwa-Mills, Lal Rawal, Christabel Enweronu-Laryea, Matilda Aberese-Ako, Kodjo Senah, Britt Pinkowski Tersbøl

**Affiliations:** c1 Ghana Health Service, Central Region, Ghana; c2 Global Health Section, Department of Public Health, University of Copenhagen, Denmark; c3 School of Health Medical and Applied Sciences, CQUniversity, Sydney, Australia; c4 Translational Health Research Institute, Western Sydney University, Sydney, Australia; c5 Department of Child Health, University of Ghana Medical School, Accra, Ghana; c6 Institute of Health Research, University of Health and Allied Sciences, Ghana; c7 Department of Sociology, University of Ghana, Accra, Ghana

**Keywords:** Healthcare-associated infections, infection prevention and control, neonatal intensive care unit, health communication, Ghana

## Abstract

Healthcare-associated infections (HAIs) remain a serious threat to patient safety worldwide, particularly in low- and middle-income countries. Reducing the burden of HAIs through the observation and enforcement of infection prevention and control (IPC) practices remains a priority. Despite growing emphasis on HAI prevention in low- and middle-income countries, limited evidence is available to improve IPC practices to reduce HAIs. This study examined the perspectives of healthcare providers (HPs) and mothers in the neonatal intensive care unit on HAIs and determined the major barriers and facilitators to promoting standard IPC practices. This study draws on data from an ethnographic study using 38 in-depth interviews, four focus group discussions and participant observation conducted among HPs and mothers in neonatal intensive care units of a secondary- and tertiary-level hospital in Ghana. The qualitative data were analysed using a grounded theory approach, and NVivo 12 to facilitate coding. HPs and mothers demonstrated a modest level of understanding about HAIs. Personal, interpersonal, community, organizational and policy-level factors interacted in complex ways to influence IPC practices. HPs sometimes considered HAI concerns to be secondary in the face of a heavy clinical workload, a lack of structured systems and the quest to protect professional authority. The positive attitudes of some HPs, and peer interactions promoted standard IPC practices. Mothers expressed interest in participation in IPC activities. It however requires systematic efforts by HPs to partner with mothers in IPC. Training and capacity building of HPs, provision of adequate resources and improving communication between HPs and mothers were recommended to improve standard IPC practices. We conclude that there is a need for institutionalizing IPC policies and strengthening strategies that acknowledge and value mothers’ roles as caregivers and partners in IPC. To ensure this, HPs should be better equipped to prioritize communication and collaboration with mothers to reduce the burden of HAIs.

**KEY MESSAGES**

- Reducing the burden of healthcare-associated infections is a responsibility for both healthcare providers and caregivers. Thus, there is a need to improve communication and interaction between healthcare providers and carers towards achieving this goal.
- Infection prevention and control in health facilities can be more effectively observed if health facilities provide the needed resources to enable health providers to undertake such measures.
- Regular monitoring and supervision in the wards can contribute to proper infection prevention and control, which will help to reduce healthcare-associated infections, especially in the neonatal intensive care units of the various hospitals.
- Pre- and post-training of health workers in health training institutions and hospitals on infection prevention and control can contribute to health providers inculcating the positive habit of strict observation of infection control measures at work. This can help to reduce healthcare-associated infections in the wards, including in neonatal intensive care units.

## Introduction

Healthcare-associated infections (HAIs) are the most frequent adverse event in healthcare delivery worldwide, and constitute a serious and preventable threat to patient safety (Nejad *et al.*, 2011; Rothe *et al.*, 2013). They lead to increased use of antibiotics, increased healthcare costs, longer hospital stays and higher morbidity and mortality rates (Gupta *et al.*, 2011; Umscheid *et al.*, 2011; Schmier *et al.*, 2016). Increased length of stay associated with HAIs varies between 5 and 30 days in low- and middle-income countries (LMICs) (WHO, 2011). Costs associated with HAIs vary from ∼US$865–US$13 000 as individual costs for various HAIs in LMICs (Pada *et al.*, 2011; Ha and Ha, 2012) to overall costs of €7 billion annually in Europe (WHO, 2011). A study conducted in a tertiary hospital in Ghana reported that the HAI treatment cost an additional US$1985 per patient, and those patients with HAIs paid twice as much as those without HAIs. The estimated annual cost of an HAI to the hospital was ∼US$700 000 and it cost the broader society almost US$900 000 (Fenny et al., 2020).

The risk of developing HAIs in health facilities in LMICs is higher than in high-income countries (Rothe *et al.*, 2013). A study by Labi *et al.* (2019) reported that the overall prevalence of HAIs among hospitalized patients in Ghana was 8.2% (range: 3.5–14.4%). Patients in the intensive care unit tend to have a higher prevalence of HAIs than those admitted to other units of the hospital (WHO, 2011). The neonatal intensive care unit (NICU) can pose a higher threat to patient safety due to its unique complexities, and factors such as understaffing, limited resources and ineffective organization of service delivery, which undermines the quality of care in LMICs (Allegranzi *et al.*, 2011; Samra *et al.*, 2011; Enweronu-Laryea *et al.*, 2018).

The burden of HAIs can be reduced through appropriate infection prevention and control (IPC) interventions and adherence to hand hygiene (HH) practices (Umscheid *et al.*, 2011; Rothe *et al.*, 2013). Compliance with HH guidelines among healthcare providers (HPs) is ∼38.7% (WHO, 2009), and ranges from 9.2% to 57% among doctors and 9.6% to 54% among nurses in Ghana (Yawson and Hesse, 2013).

The Ministry of Health (MOH), Ghana, has taken steps to improve IPC by introducing the National IPC Guidelines (MOH, 2015), manuals and protocols to improve the quality of care (Escribano-Ferrer *et al.*, 2017). The IPC guidelines provide information to HPs on standard precautions including HH, use of personal protective equipment (PPE), environmental cleanliness and waste management. These guidelines aim to promote excellence in client-centred care and maximize protection against infections for HPs and clients (MOH, 2015). Strategies aimed at reducing HAIs are however centred mostly on HPs, with little consideration for the role of patients’ relatives (caregivers). There are no clear policy guidelines on the roles of caregivers, who contribute to the basic care of hospitalized patients (Agbenyefia, 2017). Role ambiguity between HPs and caregivers could lead to frustrations in the hospital environment, where caregivers are unclear about patients' needs and do not know whether they are contributing positively to care (Ricciardelli, 2012; Agbenyefia, 2017; Alshahrani *et al.*, 2018).

The participation of caregivers in IPC promotion has been recommended as a promising strategy and there has been a call for increased caregiver engagement, which is critical to patient safety (Agbenyefia, 2017; Alshahrani *et al.*, 2018). Meaningful caregiver involvement in the joint endeavour of preventing infections is complex, and potential barriers reported by caregivers include feelings of inadequacy, fear of the consequences, having different beliefs from HPs and a desire to avoid interrupting busy HPs (Jackson *et al.*, 2003; Latour *et al.*, 2010; Rainey *et al.*, 2015; Sutton *et al.*, 2015). Caregivers may feel unprepared for IPC roles due to lack of training and little guidance from HPs (Reinhard *et al.*, 2008; Sapountzi‐Krepia et al., 2008). It is therefore critical for HPs to support the role of mothers as their babies' primary caregivers (Nyqvist and Engvall, 2009). Healthcare systems are increasingly involving families as partners, and many NICUs now promote family-centred care (Reinhard *et al.*, 2008; Sapountzi‐Krepia et al., 2008; Skene *et al.*, 2016; Ottosen *et al.*, 2019).

Some studies in high-income countries have explored family-centred care and the perspectives and roles of caregivers (Rainey *et al.*, 2015; Ottosen *et al.*, 2019; Sutton *et al.*, 2019), but such studies are sparse in sub-Saharan Africa. Studies in Bangladesh, Indonesia and South Korea have shown that recognition of caregiver involvement in IPC strategies was not included in the national guidelines (Horng *et al.*, 2016; Park *et al.*, 2020). Some studies in Ghana have explored mothers' experiences in the maternity and NICU wards (Yevoo *et al.*, 2018; Dugle *et al.*, 2020; Lomotey *et al.*, 2020) and HPs’ perceptions of the quality of neonatal care (Elikplim Pomevor and Adomah-Afari, 2016). These studies, however, were not focused on the interactions between HPs and mothers in NICUs and did not explore HAIs. Other studies addressing HAIs in NICUs in Ghana were mostly focused on incidence, epidemiology and mortality (Annan and Asiedu, 2018; Labi *et al.*, 2018, 2020).

In the face of resource and financial constraints in LMICs, infection control is a cost-effective intervention that will decrease morbidity and mortality by reducing HAIs in NICUs (Srivastava and Shetty, 2007), where mothers are important stakeholders. Furthermore, the recent COVID-19 pandemic has prompted concerns about adherence to IPC guidelines (Houghton *et al.*, 2020), hence a need to examine factors contributing to IPC compliance, to help identify strategies that will support caregivers and HPs observe IPC practices at such a critical period in global healthcare.

Local data are critical for developing and implementing evidence-based context-appropriate guidelines and protocols for IPC. To provide data to guide local policy on IPC practices, this hospital ethnographic study examined the factors that influence caregiving in the NICU, how lay mothers negotiate their roles with health professionals within the hospital context and how these interactions influence the practice of IPC and the reduction of HAIs.

## Materials and methods

### Study setting

Ghana currently has 162 district-level hospitals, 10 regional-level hospitals and five teaching hospitals (tertiary-level) in the public health sector. District hospitals form the first referral point from health centres and polyclinics, regional hospitals form the secondary-level referral point and teaching hospitals provide complex tertiary-level care (Nsiah-Asare, 2017). This study was conducted in the NICU of a tertiary-level hospital (TH) and a secondary-level hospital (SH) in Southern Ghana. The study was conducted within the context of a larger hospital-based project investigating Healthcare-Associated Infections in Ghana (HAI-Ghana project).

In this article, we present the findings of a cross-sectional study that focuses on perspectives and practices on HAI among HPs and mothers. The study sites, TH and SH, provide similar levels of neonatal care (including intravenous infusions, parenteral medicines and neonatal resuscitation). TH and SH cater to the medical needs of babies in populations of ∼5 million and 3 million, respectively. TH was selected to provide insight in the context of a larger facility, while SH provided insight from the perspective of a secondary-level health facility. The NICU of TH has a nominal capacity of 60 cots, warming platforms and incubators, and that of SH has a nominal capacity of 30. Most of the babies admitted to both NICUs are preterm and critically ill babies. The average HAI prevalence at the hospitals was 10.2% (Labi *et al.*, 2019).

TH employs ∼12 doctors, 40 nurses and other technical staff in the NICU, while the NICU in SH has around five doctors and 20 nurses. Work is organized around a similar shift pattern in both hospitals, with three shifts running (morning, afternoon and night).

### Conceptualizing the study

In this study, we consider the hospital an organizational cultural environment, with HPs, who have the biomedical and technical knowledge, and mothers from the wider Ghanaian cultural environment (Assimeng, 1999). Thus, differences and similarities in context, norms and rules are bound to conform and clash. HPs with medical expertize execute their roles while interacting with mothers in often stressful situations and this can create a huge cultural and communication chasm (Ruben, 2016).

Medical professionals have jealously guarded their exclusive rights to medical knowledge from time immemorial, and have therefore used their professional status to exclude others who do not belong to the group (Freidson, 1988). In the clinical encounter, the major concern of HPs is to ensure clinical care. The caregiver who has a highly personal and emotional involvement in the child’s illness may hold different perspectives due to the lack of technical expertize (Jones and Jones, 1975). So, whereas for HPs, infections and morbidity may just be passing clinical events, these have consequences that resonate beyond the medical realm for mothers, who are critical stakeholders. This oppositional view sets the stage for the ‘clash of cultures’. The lack of trust between HPs and mothers mainly stems from these opposing views.

Coe (1970) explains how the communication process, which is core to the medical encounter, is asymmetric between HPs and caregivers. This inherent asymmetry is reinforced by the medical profession’s socialization practices which ground a relationship of domination, reflected in the management of information exchange and utilization of medical jargon (Filc, 2006). In the absence of clear lines of communication, anxious caregivers produce their own scripts to assuage their anxiety over the health status of their patients (Senah, 2002). Caregivers often accept these patriarchal attitudes of HPs without questioning, as a reflection of the broader society, where questioning authority is perceived as insubordination (Zaman, 2004). Communication between HPs and caregivers can lead to an improvement in several areas of health and well-being, while the lack of communication or poor communication could result in poor compliance with guidelines (Jones and Jones, 1975).

Under a broader paradigm of sociocultural theory, Bourdieu (1991) emphasizes that when individuals interact, they do so in a specific social context, ‘the field’, which shapes their practices, perceptions and attitudes. Social fields in medicine include the field of health provider–client interactions (Emmerich, 2013), where positions of power are determined by medical knowledge, professional prestige, etc. Power is present in all interpersonal relationships, is relevant in healthcare and ‘comes into being’ when it is put into action through ‘strategies’ such as expressions through language and communication (Foucault, 1982).

## Study design

We used an ethnographic approach involving qualitative interviews with mothers and HPs, participant observation, informal meetings and discussions. An ethnographic approach allows us to obtain rich details of social phenomena, and requires long periods in the field to actively study, experience and represent the lives of participants in their natural setting (van der Geest and Sarkodie, 1998; Emerson *et al.*, 2011). In-depth interviews and focus group discussions were used to collect data.

### Selection of study participants

Women 15 years and older, whose babies had been hospitalized in the NICU for a minimum of 48 h during the study period, were eligible to participate in the study. Purposive sampling was used to recruit mothers to share their perspectives and experiences of care in the NICU. A total of 22 mothers participated in the in-depth interviews, and 24 mothers participated in four focus group discussions with between four and eight mothers per group: (TH: 15 interviews and two focus groups [*n* = 12]; SH: seven interviews and two focus groups [*n* = 12]). Sixteen HPs participated in the in-depth interviews. A cross-section of frontline HPs, health managers and IPC coordinators were purposively selected to achieve diversity in terms of staff cadre and level of experience.

## Data collection tools

Qualitative interview guides were developed to capture nuanced contextual information related to the topic. A range of relevant literature was reviewed to develop the question guides consisting of semi-structured questions and probes to address the objectives of the study. The question guides were slightly revised for clarity, comprehensiveness and relevance, following a pilot test with three mothers and three HPs in a similar ward setting who were not included in the study.

A health facility checklist was developed based on the review of the WHO ward infrastructure survey and existing literature (WHO, 2009; Yawson and Hesse, 2013) to support data collection. The checklist captured the available HH facilities on the wards. Both the qualitative interview guides and checklists were developed in English.

### Data collection

The ethnographic study was conducted in both hospitals between January and June 2018. Interviews lasted 45 min to one hour and the focus group discussions lasted one hour to an hour and a half. Interviews were conducted face-to-face in the hospital in a calm location as per the convenience of participants, and participants were interviewed alone. Demographic information was collected, and participants spoke about their experiences of IPC practices and interactions in the ward.

The first author, GSM, conducted interviews with HPs in the English medium. GSM is a medical doctor and a PhD student experienced in qualitative research. GSM understands issues related to caregiving and used her student–researcher identity to observe care and interactions in the ward. Two trained female research assistants who were graduate students in health-related fields and with qualitative research experience assisted with data collection. The researchers were not familiar to the participants prior to the study. All researchers were fluent in the local Akan language.

Some mothers’ interviews were conducted in English and others in Akan by GSM and the research assistants. GSM conducted the FGDs, while a research assistant took observational field notes. Participants were able to freely speak English or Akan, so they could express themselves comfortably. Probes were incorporated, but participants did not need much prompting to share their experiences and ideas. Participants were offered refreshments after the interviews. The interviews were audio-recorded, transcribed verbatim and those in Akan were translated into English by a research assistant. The translation was verified by a second research assistant. Data saturation was achieved. Field notes were documented, reconstructed and expanded following each ward visit, and data were incorporated into further ethnographic analyses (Emerson *et al.*, 2011).

More than 100 h of participant observation and informal interactions with mothers and HPs were also conducted, to observe activities and interactions relating to IPC and HH. Personal HH practices observed included HH upon arrival and before leaving work at the end of the day or duty shift. Quantitative data on HH compliance (WHO, 2009) were captured and are reported elsewhere.

### Data processing and analysis

Transcripts and notes were kept confidential in password-protected files. Transcripts were read by two team members (GSM, BPT) to develop a coding structure reflecting issues arising from the data. Coding of the transcripts was done using NVivo, and commonly occurring themes and subthemes were identified. We read and re-read the transcripts for the overall experiences being presented and coded to develop open, focused and theoretical codes to describe dimensions of participants’ experiences and interactions. This study takes a constructivist epistemological approach, where knowledge is dependent on perception and experience, and drew on inductive thematic coding, memo writing and reflexivity (Charmaz, 2001).

We used a grounded theory approach for data analysis. This enables in-depth exploration of multiple subjective experiences, provides explicit, sequential guidelines for conducting qualitative research and helps the researcher to streamline and integrate the data collection and data analysis process. Grounded theory allows the results to be ‘grounded’ to the data collected (Glaser *et al.*, 1968; Chun Tie *et al.*, 2019). The preliminary findings of the study were presented to HPs and managers in both hospitals in seminars to return the results for verification and validation of the study. Comments and suggestions received during the seminar were incorporated into the final report.

**Figure 1:**
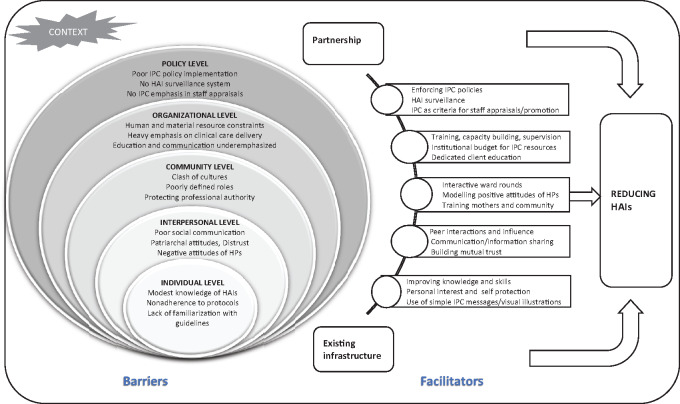
Conceptual framework illustrating barriers and facilitators to reducing HAIs in Ghana. Adapted from the SEM of healthcare

The presentation of our findings was guided by the Social Ecological Model (SEM). SEM has been recognized and accepted for use broadly in the efforts of enhancing health and well-being, and is widely used to better understand the health behaviours of individuals. SEM acknowledges that an individual’s behaviour is shaped through multilevel factors. In general, five hierarchical levels of SEM have been recommended and used in social science, psychology and health science sectors: individual, interpersonal, community, organizational and policy levels (Newes-Adeyi *et al.*, 2000; Lounsbury and Mitchell, 2009; Rawal *et al.*, 2020). Themes denoting factors influencing the practice of IPC to reduce HAIs were categorized under the various SEM levels, and other arising themes were also integrated (Figure 1). SEM offers a holistic understanding of the factors influencing IPC practices of HPs and mothers.

Strategies were employed to ensure the trustworthiness of the findings, such as checking the data for accuracy and completeness and using team meetings to establish coding consensus (Shenton, 2004; Houghton *et al.*, 2013). Reflexivity was employed, such as GSM taking note of her own preconceived ideas as a medical doctor which could influence observations in the study settings. Researchers examined personal biases and the effect of the researcher on the research process and interpretation of findings. We used the COREQ approach (Tong *et al.*, 2007) to report on the characteristics of the research team, study design, data collection, data analysis and other strategies (Supplementary File).

Ethical clearance was obtained for this study (GHS-ERC 07/03/2017). Written informed consent was obtained from interview participants who were informed about the study objectives, and all ethical procedures were followed. For confidentiality, direct quotes from participants are identified by codes (Doctor, D; Nurse, N; Manager, MG; Mother, MT; PA, Physician Assistant).

## Results

HPs who participated in the study included six males and 10 females, aged 21–60 years. There were eight HPs from each hospital, including two managers, two IPC coordinators, four doctors, one physician assistant and seven nurses, with 50% of HPs having worked five years or less in the NICU. Mothers were between the ages of 15 and 49 years, and >50% of them had secondary school education or higher. Table 1 presents the characteristics of mothers in this study.

**Table 1:** Characteristics of mothers who participated in the study

Demographic characteristics	Number	(%)
Age		
15–19	4	9
20–29	19	41
30–39	22	48
40–49	1	2
Marital status		
Single/other	12	26
Married	34	74
Education		
None	3	7
Primary	19	41
Secondary	10	22
Tertiary	12	26
Postgraduate	2	4
Number of days on admission		
<14	31	67
15–28	11	24
>28	4	9
Baby’s diagnosis (*N* = 56)		
Prematurity	35	62.5
Other	21	37.5

### Individual-level factors influencing IPC practices 

#### Knowledge of HPs and mothers on the relevance of IPC

All HPs agreed that knowledge of HAIs is important, and that promoting IPC in the ward is critical (Figure 1, Individual-level factors). HPs described a clear association between observing IPC measures and reducing HAIs. HPs mentioned HH, waste segregation and disinfection as some measures to reduce HAIs. The national IPC policy document was not available in the wards, although there were clinical protocols and HH messages on posters in both NICUs. During an observation session, nurses were seen debating which detergent to use for cleaning incubators (Observation#21TH), and this was later clarified by a manager who said the information was in the ward protocol. The manager stated: ‘People should familiarize themselves with and use the protocols and posters’*.* The manager also complained that knowledge of HAIs among staff was low.

Mothers were aware of the possibility of acquiring infections in the NICU and described infections with terminologies in Twi such as ‘*mmuawa*’ (germs) and ‘*yareɛ/yadeɛ*’ (diseases). Mothers were observed washing their hands before entering the NICU, which they explained was to avoid contaminating their breastmilk or transferring germs to their babies. A mother said: ‘Babies are quite delicate; their immune system is not built to term’. Mothers said they heard about ‘infections’ through talks at antenatal clinics, and from the television and radio. A mother whose baby had previously acquired an HAI mentioned that she was motivated to wash her hands to avoid expenses associated with HAIs:

*With my first child I didn’t listen to what the nurses said, I was stubborn. So, I had to keep visiting the hospital several times. This time, I wash my hands and do exactly as I am told so that no disease will affect my child. Yes, because right now, there is no money* (MT9).

#### Attitudes of HPs and mothers towards IPC

HPs did not strictly follow protocols and were seen using their discretion during some procedures (Figure 1, Individual-level factors). A nurse who was observed administering medications with only one gloved hand explained that she was ‘saving gloves’, as gloves were scarce on the ward (Observation#24TH). Some senior nurses felt that student nurses who used gloves while changing soiled cot sheets were wasting the limited supplies. Some HPs also used normal examination gloves and sterile gloves interchangeably, although there were guidelines for the use of each type of glove.

HPs who were observed performing HH used soap and water stored in *veronica buckets* (improvised buckets with a tap) (Veronica Bucket, 2020) when running water was not available in the ward. In both NICUs, <50% of observed HPs performed the recommended hand washing steps correctly, according to WHO guidelines. However, HPs in TH were more compliant with HH and use of the alcohol hand rubs than HPs in SH (Table 2).

**Table 2:** HH practices of HPs in two NICUs in Ghana, as observed and compared with effective techniques recommended by the WHO, January 2018

	Soap and water used for HH	Running water available for HH	Handwashing for 40–60 s	Hands cleaned with alcohol hand rub	Staff dry hands with clean single- use towels	Performed steps of hand washing appropriately	Total (%)
THC1	✓	✓	✓	✓	✓	0	5 (83)
THC2	✓	0	0	✓	0	0	2 (33)
THC3	✓	✓	0	✓	0	0	3 (50)
THNS	✓	✓	✓	0	✓	0	4 (67)
SHC1	✓	0	0	0	0	0	1 (17)
SHC2	✓	✓	0	0	0	0	2 (33)
SHNS	✓	✓	✓	0	✓	0	4 (67)

✓ = >50% of HPs complied with HH procedures.

0 = <50% of HPs complied with HH procedures.

C1, C2, C3, cubicles 1, 2 and 3; NS, nursing station; SH, secondary hospital; TH, tertiary hospital.

HPs referred with concern to instances of babies acquiring HAIs from other babies especially when two or three unrelated newborns share an incubator for warmth or a cot for phototherapy. A mother narrated how one of her twin babies got an HAI from the other twin:

*I asked the doctor… but they are sleeping in the same incubator…they touch each other? She said… ‘ok yes, ideally, we should have moved one out when we realized the other had an infection’… I was like… let’s do the test on the other one and see if he’s fit. They did the test and he had also picked up the infection. So now they are both on the Vancomycin* (MT5).

#### Beliefs of HPs and mothers about IPC

Many HPs focused on protecting themselves from HAIs and mentioned that they did not want to take any infections from the hospital to their homes. HPs reported performing HH because of fear of becoming infected themselves. One HP said: ‘I think what motivates most people, including myself, is not cross-infection. It is always about personal protection’.

Some HPs perceived that mothers were not interested in IPC. However, mothers did observe and scrutinize IPC practices among HPs, and formed ideas and clues about IPC from what they saw. Mothers mentioned that they observed HPs wearing gloves to protect themselves and the babies from infections. Mothers described their own habits of handwashing before eating, after using the bathroom and before touching their babies.

#### Skills of HPs and mothers on IPC

There were more specialist paediatricians and neonatal nurses in TH, and there was frequent training on neonatal care for the HPs. TH had a full-time IPC nurse who was focused on IPC in the wards, while SH had an IPC nurse who had to fulfil other clinical roles. IPC training had been conducted for HPs who had worked in the NICUs for more than a year, but not for HPs and students who joined the wards subsequently or for short rotations. Doctors were generally responsible for invasive procedures like insertion of intravenous cannulas, but nurses also performed these procedures in SH.

Seven per cent of mothers had no formal education at all, while 41% of mothers had attended school up to the primary level (Table 1), but most were not able to understand or read English well. This presented a challenge with the use of written media as a channel of IPC communication, as HH posters and instructions were mostly in English. Mothers expressed the desire to receive IPC information via simplified messages on social media, using visual illustrations and/or in the local languages (Figure 1, Individual-level factors). Some mothers reported that they were not instructed about HH in the NICU. One mother said: ‘Nobody has taught us how to wash our hands, so we just wash them anyhow’.

### Interpersonal-level factors influencing IPC practices 

#### The influence of peers on IPC practices

HPs mentioned that they are often inspired or reminded by colleagues to perform HH (Figure 1, Interpersonal-level factors). One HP mentioned that ‘some people will take reminders in good faith and change their behaviour; some will not’*.* Our observations in TH showed that HPs had a practice of engagement in shared breaks and meals in a staff room in the NICU (Observation#23TH). This provided an environment where HPs interacted and shared information on a range of topics, including IPC.

Some mothers said that they acquired knowledge of HH by watching other mothers. Mothers held conversations in the waiting areas and exchanged knowledge by sharing experiences.

#### The influence of communication on IPC practices

HPs argued that due to the high turnover of mothers and babies, it was not possible to provide education on IPC to every mother, although most mothers received some orientation. With reference to Figure 1, communication on the interpersonal level is very crucial to the reduction of HAIs. However, from our observations, communication between HPs and mothers was limited. Mothers craved care-related information, and expected explanations regarding decisions taken concerning their babies. A mother was told that ‘we tell you only what is important for you to know’ when she approached a doctor for information on her baby. Another mother complained that the doctor was ‘more interested in laboratory tests which were to be done for the baby’. Although laboratory tests are an essential component of clinical care, the mother wanted a social discussion while the HP approached it from a biomedical perspective.

#### HPs’ patriarchal style of communicating with mothers and distrust of mothers’ ability to comprehend IPC

Interactions between HPs and mothers were more of a one-way dialogue when they occurred, with HPs instructing the mothers on what to do, or mothers reporting back to HPs on specific issues. HPs mentioned that some of the mothers were not competent enough to process technical information about IPC. Some HPs mentioned that some mothers do not know how to use sinks and toilets, because they were brought up in villages where such facilities are mostly unavailable, and that these mothers have also not been trained on hygiene practices. One HP stated: ‘some of them are villagers…it is the way they are trained’. Some HPs perceived mothers as potential sources of infections. A nurse stated: ‘they sit on the floor on the corridor downstairs, then come here with the dirty cloth’.

Some HPs mentioned that mothers or their relatives were capable of sneaking in herbs to be applied to the babies’ umbilicus, contrary to the hospital protocol of using chlorhexidine, which was not known to most mothers. During FGDs, mothers discussed the use of spirit, gel or local herbs on their babies’ umbilical cords as part of traditional newborn protective care. A mother complained:

*I came here last night and up till now they haven’t given me spirit to clean the baby’s navel, so this can also cause infection* (MT7).

Mothers also discussed that in caring for their babies, they sometimes received conflicting messages because ‘nurses will say one thing, and grandmothers will say something else’.

### Community-level factors affecting IPC practices

Ward rounds were regularly conducted as part of the routines in both NICUs, where HPs would give a detailed account of each baby to a supervising consultant who would then lead an academic discussion. Ward rounds represented an avenue for interaction where shared values on IPC came into play (Figure 1, Community-level factors). We observed that HPs were more likely to perform HH during ward rounds when the leading consultant paid attention to HH (Observation#22TH, #39SH).

Mothers had no role in ward rounds, and HPs preferred that they would be absent during this period to avoid interfering with questions and unsolicited comments. Mothers were termed ‘difficult’ if they did not comply. Mothers sometimes needed more time to complete care activities. One mother said:

*When leaving NICU you are tired and frustrated because you did not finish feeding your baby, and you are wondering if the nurses will continue feeding them for you or not* (MT22).

There are restrictions on visits by family and friends in both NICUs. Staff explained that this was an IPC measure. However, the reason for this restriction was not understood by most mothers. These mothers were opposed to these restrictions, as the local culture encourages family members to celebrate the arrival of a new baby by seeing the newborn. One complained:

*My sister came all the way from Cape Coast (Central region) but she hasn’t seen the baby yet… nobody else has seen the baby, which is worrying* (MT14).

A mother complained that when she tried to get a nurse's attention to see to her baby, she was told casually that ‘as for pre-terms their condition can change at any time’. A manager later explained how some cultural beliefs reflect in HPs’ attitudes towards the babies:

*Culturally people think the babies still belong to the spirit world before day seven… the way they start treating them changes afterward; less effort is needed to convince staff to go the extra mile after day seven* (MG1).

### Organizational-level factors influencing IPC practices

#### Human and material resource deficits that affect commitment to IPC

Managers emphasized the need to have the necessary human and material resource allocations for optimal IPC practice (Figure 1, Organizational-level factors). HPs expressed the need for more HH stations and supplies in the NICU. Some HPs mentioned that due to the scarcity of PPE such as aprons, masks and boots, some PPE intended for single use are used multiple times. The NICUs lack an adequate supply of gloves, and HPs sometimes had to borrow gloves from other cubicles and wards or improvise by using a single glove at a time rather than a pair. On other occasions, HPs wore double pairs of gloves, explaining that they could not trust the quality of some types of gloves. Staff purchased and brought their own scrubs (PPE) to work and were responsible for cleaning them.

HPs reported on the struggle to make IPC a priority because of clinical demands, including several new admissions daily and time-consuming clinical responsibilities. A doctor in SH said: ‘A doctor's work is clinical care, and if the clinical workload is heavy, sometimes it's only natural that you'll overlook other things’.

The health facility checklist was used to assess ward infrastructure. The NICU in TH had three cubicles, with two sinks which were not always functional. SH had a much smaller NICU space, with two cubicles with a sink in each. Neither of the NICUs had a steady supply of soap, water or towels at the sinks, and the sinks designated for handwashing by mothers generally had the least supplies. HPs sometimes had to move out of the cubicles to the nursing station to access a sink with running water, and this was reported as a barrier to HH compliance (Table 3).

**Table 3:** Observation of ward infrastructure for HH using Health Facility Checklist

Room no./ID	Total no. of beds/cots	Beds with AHR within arm’s reach	No. of sinks	No. of sinks with clean water	No. of sinks with soap	No. of sinks with disposable towels	No. of sinks with clean water, soap and disposable towels	Total no. of AHR dispensers in this area	No. of fully- functioning and filled dispensers	No. of HPs encountered	No. of HPs encountered with AHR bottle in their pocket
THC1	18	0	2	1	1	1	1	4	2	8	0
THC2	15	1	2	1	1	1	1	3	2	6	0
THC3	22	2	2	2	1	0	0	2	1	8	0
THKMC	5	1	3	3	3	1	1	1	1	2	0
SHC1	10	0	1	1	1	0	0	0	0	7	0
SHC2	20	0	1	1	0	0	0	0	0	3	0
Corridors or other areas with points of care											
THNS	0	0	1	1	1	1	1	1	1	3	0
SHNS	0	0	1	1	1	0	1	0	0	1	0
THM1	0	0	1	1	0	0	0	0	0	0	0
SHM1	0	0	1	1	0	0	0	0	0	0	0

Point of care: the place where three elements occur together—the patient, the healthcare worker and care/treatment involving contact with the patient and their surroundings.

AHR, alcohol hand rub; C1, C2, C3, cubicles 1, 2 and 3; HPs, health care providers; KMC, kangaroo mother care, where mothers are roomed in with their preterm babies to do skin-to-skin nursing; MS, mothers' HH area (at entrance); NS, nursing station (for use by HPs only); SH, secondary hospital; TH, tertiary hospital.

One HP complained:

*We use kitchen liquid soap to wash our hands, and it is so diluted. Disinfectant for cleaning equipment is also so diluted that it’s meaningless. So when we clean the incubators, we’re only redistributing the germs, because we don’t have the right disinfectants* (D2).

### Policy-level factors affecting IPC practices

Although a national IPC policy exists, it is not applied optimally in the NICUs. Only a few HPs said they had seen it previously or attempted to read it (Figure 1, Policy-level factors). HPs wanted soft copies or summaries of essential practical portions of the bulky guideline in the form of posters or smaller protocols that can easily be assessed and utilized on the wards.

There was no structured HAI surveillance in the NICU wards, so HPs were not aware of or able to keep track of HAI rates. Although both hospitals had microbiology laboratories that could identify infectious agents to treat babies who develop HAIs, mothers had to bear the costs of this, which some could not afford.

#### Training and supervision to improve IPC practices

Managers mentioned that there is a need for supervision to improve adherence to IPC guidelines in the wards. Managers also mentioned that senior nurses should enforce policies by being on the frontlines to work with the HPs and supervise them. As one manager said, ‘If you don’t war with them, you can’t tell them how to fight’ (MG2).

Managers suggested the need for regular IPC training, which should not only be about the technical aspects of HAIs but should also cover the basics such as communication with clients. One said: ‘Medical students should be taught to respect patients and relatives, even before they graduate’. Managers in both hospitals expressed the need to have a team of dedicated staff to oversee IPC activities and make IPC teams fully operational. Staff also undergo yearly appraisals; however, IPC is not a critical part of this appraisal. A manager mentioned that it would be useful to include IPC compliance as part of the criteria for staff appraisals and promotions (Figure 1, Policy-level factors).

#### Partnership to improve IPC in the NICU

Mothers or family members are required to convey specimens to the laboratory, retrieve laboratory results and arrange the purchase of medicines for their babies. A partnership is crucial in this context because if mothers failed to fulfil this expectation, treatment could be delayed. Sometimes, mothers delayed in providing funds for laboratory tests due to a lack of understanding of the need for these tests in diagnosis (Observation#41SH). A mother explained that she would rather prioritize the use of her funds to buy medicine to cure her baby’s illness than for tests that provide no cure.

Mothers perceived partnership as HPs being present and attentive in interacting with them and their babies. Mothers and nurses performed common care activities for babies such as bathing, changing nappies and feeding. Mothers of preterm or low-birth-weight babies practised ‘skin-to-skin’ or kangaroo mother care in assigned rooms, which offered the opportunity to interact with HPs, especially in TH where an assigned nurse was present during the day shift. Nurses who were perceived to be friendly and showed positive attitudes towards mothers became the preferred ones whom most mothers would approach for interactions.

Mothers received instructions from nurses on care practices, but this was often circumstantial and unstructured. Mothers referred to specific behaviours such as frowning, which made them feel unwelcome to interact with some HPs. Mothers expressed the need to be treated with respect irrespective of their background, as some felt that they were probably older than some HPs, which is especially a factor in Ghanaian society where seniority by age is given cognizance. A mother said: ‘We are all human beings…so you should treat us as sisters…or friends’.

When researchers enquired whether mothers would like to have an IPC-related role such as reminding staff to wash their hands, the responses suggested that mothers felt reluctant to participate in such roles. Mothers indicated that they did not want to be perceived as interrupting the provision of care. A mother said: ‘When you try to do that, they will tell you that you are trying to tell them how to do their job’. Another mother who happened to be a doctor also said:

*As soon as you wear a patient’s coat, you become a patient… so sometimes, you wouldn’t want to offend the one taking care of your baby, because you feel that this person is taking care of my baby and what if she leaves my baby?* (MT2).

However, some mothers said they recognized the need to assume more responsibility to protect their babies. A mother who had lost one of her twins following an HAI believed it was the fault of the HPs and blamed the NICU practice of nursing twins together in the same incubator. She had concerns about the safety of the second twin and stated:

*If the one on duty is not doing well with my baby, I will complain; even if the person gets annoyed, I don’t care!* (MT6).

One outspoken mother mentioned that some of the nurses found her irritating for being rather assertive:

*I mean once I find out the thing is not properly handled, I am not going to tolerate that, so they felt that I was irritating* (MT8).

HPs perceived that their authority would be challenged or mothers would lose confidence in them if mothers were empowered to take up the role of reminding them about HH. Some direct quotes reflecting HPs’ perceptions are captured in Table 4. HPs mentioned that it would require resources to promote IPC among mothers, e.g. the provision of aprons for mothers’ use, to minimize infection risks to the babies. HPs added that they needed mothers to be compliant in caring for their babies. One HP said:

*When we are fortunate to get ‘correct’ mothers who know how to feed and handle their babies properly, the burden goes down…they pay for their labs and they are here to show love to their babies* (N8).

**Table 4:** Direct quotes by HPs on perceptions of caregiver involvement in IPC in the NICU

*When you give them [mothers] that chance, the way they will talk to you might provoke you…but we as nurses should know what we are about* (N1).
*It will bring problems because some of the mothers might think they know better than us… They will never have confidence in you. They will think you don’t know your job… It brings down your morale* (N2).
*Some of them…they are rude. It depends on how you will talk to me. If you are trying to teach me my work, then I will also give you what you also don’t know* (N3).
*In our setting, it will be difficult for a patient to tell a nurse to wash their hands…probably a health worker will feel offended when a patient says something like that, unless maybe it is said in a playful way (*D1).
*It will be beneficial to them in the long run because when they go home it will also help them* (N6).
*I think it will also help you the health worker when they draw your attention to these things to keep you in check* (N7).
*If it is true you haven’t washed your hands then you should take it cool. I don’t think there is the need for me to get upset… I don’t know about my other colleagues* (PA1).
*It’s all about continuous education. At least when we are always reminded about a precaution…we also have to involve the relatives in the education* (D2).

## Discussion

This study explored the perceptions of HPs and mothers on HAIs across a secondary and tertiary hospital in two NICUs in Ghana and provided contextual information about IPC practices. The findings of this study that HPs have modest levels of knowledge of HAIs are comparable with findings in previous studies in Ghana and elsewhere (Ocran and Tagoe, 2014; Ogoina *et al.*, 2015; Akagbo *et al.*, 2017; Wahdan *et al.*, 2019). There were key barriers and facilitators to IPC practices to reduce HAIs. The barriers include contextual factors such as resource constraints, HPs’ distrust of mothers, the negative attitudes of some HPs and HPs' fear of having their authority undermined by mothers. Facilitators included the positive and approachable attitudes of some HPs, influence from colleagues to perform IPC and mothers learning from other mothers in the NICU. This study further used the socio-ecological model to present how personal-, interpersonal-, community-, institutional- and policy-level factors interact to influence IPC practices.

While HPs reported that they gave mothers some information and orientation, they also admitted that heavy workloads and resource constraints hindered their ability to communicate IPC practices to mothers. Yet, they blamed mothers for some practices that they perceived as not hygienic enough. Mothers, on the other hand, reported that they were willing to learn hygienic practices since their interest was to protect their babies. Similarly, Aboungo *et al.* (2020) found that mothers were often blamed for negative health outcomes, and that blame is often directed at the actions or inactions of mothers or other factors that concern mothers. Blaming mothers may be a way of diverting responsibilities from HPs and other stakeholders.

Our findings revealed a cultural clash between HPs and mothers, as HPs sometimes adopted a patriarchal approach, where mothers were given instructions to comply with but were not given the opportunity to ask questions or to comment on hygiene practices. Also, HPs expressed some level of distrust of mothers’ ability to adapt to the hospital culture of hygiene practices and the use of hospital facilities, as there was the belief that some mothers came from villages that lacked such facilities and so were not familiar with their use. Similarly, Andersen’s (2004) study in a regional hospital in Ghana revealed differential treatment towards clients who were considered poor, ignorant and uneducated, summed up in the term ‘villagers’.

A barrier that hindered HPs’ communication on IPC with mothers was the fear that sharing professional and technical knowledge could compromise their authority in the NICU. HPs, therefore, minimized the exchange of information with mothers. Abraham and Shanley (1992) described HPs as ‘authority figures’ who engage in this behaviour to ensure that their authority and expertize remain unchallenged. McCann *et al.* (2008) also reported that fear of loss of power and control on the part HPs contributed to limitations being placed on parental interactions and involvement in care. Other studies have shown that HPs derive immense power from their knowledge, skills and access to clients, in the social field of the ward (Lipsky, 1980; Mintzberg, 1983).

Our findings show a gap in readiness on the part of most HPs to have empowering dialogues with mothers. Contrary to our findings, Aston *et al.* (2015) found that HPs challenged a health discourse that situated HPs as authority figures, and chose to develop relationships in ways that would build mothers’ confidence and improve partnership. Bodenheimer *et al.* (2002) differentiated strength-based discourses, focused on mothers identifying and making healthcare-related decisions, from the historical medical discourse that positions HPs as experts who assess and judge mothers’ actions and ultimately tell them what to do. This suggests a need for transformation of HPs’ perspectives and approach to dialogues with mothers in NICUs in Ghana, as the implementation of IPC practices to reduce HAIs is influenced by building and nurturing trust to improve collaboration between HPs and mothers.

Mothers, irrespective of their background, felt powerless when their babies were admitted into the NICU because this was a different cultural context. Mothers felt compelled to submit to the higher authority of HPs in the interest of receiving quality care for their babies. This reflects the wider Ghanaian society, where traditional and institutional authority is obeyed and not questioned (Assimeng, 1999). Such a practice was detrimental to mothers’ ability to communicate and seek clarification from HPs, which led to safety concerns among mothers. Also, Zaman (2004) noted that the values and norms of society are expressed in the hospital wards, where people socialize hierarchically, and caregivers, who are mainly from poor economic backgrounds, are at the bottom of the hierarchy.

The organizational culture in the NICU, which focused on biomedicine and thus dealt with technicalities and appropriate ways of holding babies or refraining from holding them as a form of IPC practice, was at variance with the typical Ghanaian culture that encourages parents and family members to hold and show affection to newborn babies. Sometimes HPs distrusted mothers and perceived them as a potential risk of infection to the babies, and this hindered positive and clear interaction between HPs and mothers. As the cultural ethos of each group presents a set of expectations and interpretations often at variance with one another, this adds new twists to the clash of cultures (Senah, 2002). The limited communication further engendered mistrust among mothers of the good intention of HPs to protect their babies. Some mothers also felt disrespected by HPs due to their language, actions or inactions. However, respect did not appear to be the focus when HPs were dealing with issues relating to quality of care. Coe (1970) described the lack of effective communication between HPs and clients as a great source of discontent in hospitals.

Mothers were comfortable interacting and engaging with HPs who exhibited positive attitudes. This suggests that mothers are interested in gaining more knowledge and collaborating with HPs in the interest of their babies’ health. However, they were also influenced by cultural beliefs and experienced a dualistic sense of responsibility to satisfy both cultural and hospital expectations when caring for their babies. This is similar to findings from previous studies in Zambia and Ghana (Moyer *et al.*, 2014; Buser *et al.*, 2020). To address the varying cultural perspectives that engender distrust between HPs and mothers, further engagement and negotiations between HPs and mothers would be beneficial. Also, grandparents and other family and community stakeholders who influence mothers’ decisions in newborn care should be engaged during educational sessions at the community level.

One barrier to hygiene practice were resource constraints, which led to improvising such as gloving one hand instead of two when there were glove shortages. This also affected the HPs’ ability to offer resources to mothers to encourage them to observe IPC practices. HPs had more access to HH resources than mothers, similar to findings in other LMICs (Horng *et al.*, 2016). Other studies have shown that when HPs are offered limited resources in the provision of health care, they exercise discretionary power by improvising and modifying policies, thereby influencing how policies are enacted (Walker and Gilson, 2004; Aberese-Ako et al., 2014). Similarly, a study in Ghana found that it was common for HPs to improvise or modify protocols when basic supplies, logistics and infrastructure needed for adherence were inappropriate or not available (Yevoo *et al.*, 2020). For HPs to be able to respond to the needs of clients, there is the need for their requirements for essential resources, supplies and infrastructure to be addressed (Enweronu-Laryea *et al.*, 2015; Aberese-Ako, 2016), as these concerns compete with their focus on HAI concerns.

This study illustrates the importance of discussing a partnership between HPs and mothers and negotiating the role of mothers. In doing this, attitudes, socio-cultural norms and the power distance wherein HPs and mothers operate in a super- and subordinate relationship (Senah, 2002) should not be overlooked. HPs should be aware of how their positions of expertize within the NICU affect interactions with mothers. A deeper understanding of personal, social and institutional aspects of IPC and HAIs will provide opportunities to reflect upon and change practices to support and involve mothers. Partnerships foster improved adherence, and ultimately improve healthcare outcomes (Martin *et al.*, 2005). Participation, engagement, negotiation and sometimes compromise enhance opportunities for interactions in which mothers, as key stakeholders, take responsibility for their part in promoting IPC to reduce HAIs.

The findings of this study showed that IPC practices have not been implemented effectively in the NICUs. These findings suggest that communication and partnership that encourage caregiver involvement in IPC should be developed through interactions. Similar to our findings, other studies have pointed out the need for the medical and nursing curricula to emphasize interpersonal communication in healthcare, and to incorporate trainings that allow HPs to learn, practise and reflect on their provision of respectful care and communication (Afulani *et al.*, 2019; Lim *et al.*, 2019). These trainings should be incorporated into the pre-service and in-service training of HPs to improve mothers’ experiences in NICUs in Ghana.

Our findings outline the current challenges associated with the effective practice of IPC, which should guide policymakers to strengthen measures to improve the implementation of existing IPC policies in the NICU. Findings from this study provide insights to inform strategies to raise the priority of IPC and limit harm from HAIs in Ghana.

### Limitations

Interviews with mothers were conducted within the hospital setting, and mothers may be unwilling to be critical of HPs who are caring for their hospitalized babies. We took steps to build trusting relationships with the mothers and to assure them of confidentiality. We also spent long periods building rapport with HPs to minimize the Hawthorne effect. There is the potential for losing meaning and nuance in the interviews which were conducted in Akan and translated into English. Transcripts were doubled checked by a second research assistant to ensure that original meanings were retained as much as possible. This study explores attitudes and general beliefs, and presented only a few examples of actual cases of HAIs. An important next step in research would be to link attitudes, beliefs and practices to the occurrence of HAIs.

## Conclusion and recommendations

HPs and mothers demonstrated a modest level of understanding about HAIs and IPC practices. Some key barriers and facilitators to knowledge and observance of IPC practices to reduce HAIs were identified. The barriers included non-adherence to protocols, negative and patronizing attitudes of some HPs towards mothers, fear of loss of authority, resource constraints in the hospital systems and poor supervision and implementation of IPC policies. Facilitators included positive and approachable attitudes exhibited by some HPs within the NICU, influence from colleagues to perform IPC, mothers receiving information on IPC from the antenatal clinics and peer support from other mothers.

There is the potential to form a partnership between HPs and mothers in promoting IPC practices in Ghanaian health facilities. This is critical considering that Ghana is a low-resource country with limited budgets for health care, so improving IPC could reduce infections and thus save facilities and families from extended hospital stays, with consequent costs of treatment. However, this requires partnerships where mothers are seen as part of the solution, for if mothers are perceived as distrustful and seeking to undermine the authority of HPs, this common objective will not be achieved.

Effective communication between HPs and mothers should be a key area of focus in promoting partnerships to reduce the burden of HAIs, particularly among neonates in Ghana. This requires clearly defined policies and strategies that define, acknowledge and value mothers’ roles as caregivers, and encourage partnership between HPs and mothers. There is a need for maintaining standard precautions and practices more effectively and efficiently. HPs need to make deliberate efforts to go beyond personal and professional barriers, to acknowledge the role of mothers in patient safety and to empower mothers and caregivers in promoting IPC.

There is a need for hospitals to improve the supervision and monitoring of HPs, as some of the gaps in IPC compliance were noted to be due to limited supervision and follow-up. In addition, there is the need for hospitals to devote more funds to providing equipment and hygiene-related medical supplies, which will help to improve hygiene conditions. Also, IPC guidelines should be made available to all staff, and training on HAIs and IPC should be provided regularly to incoming staff and students. Structured training at health facilities must aim to both provide both technical knowledge and develop HPs’ interpersonal communication skills, to help bridge the gap between HPs and caregivers. It is also important that medical and nursing curricula emphasize interpersonal communication and patient-centred care.

## Supplementary data

Supplementary data are available at *Health Policy and Planning* online.

## Supplementary Material

czaa102_Supplementary_Data
